# Expression of Concern: TET1 exerts its tumor suppressor function by regulating autophagy in glioma cells

**DOI:** 10.1042/BSR-20160523_EOC

**Published:** 2020-07-02

**Keywords:** autophagy, CRISPR/Caspase-9, glioma, methylation, TET1

The Editorial Office has been made aware of potential issues surrounding the scientific validity of this paper, hence has issued an expression of concern to notify readers whilst the Editorial Office investigates.

